# Supplementary material for: Living with the pathogenic chytrid fungus: Exploring mechanisms of coexistence in the harlequin toad Atelopus cruciger
Source: PLoS One. 2021 Jul 15;16(7):e0254439. doi: 10.1371/journal.pone.0254439 (PMC8281998; doi:10.1371/journal.pone.0254439)
Supplement: S1 Appendix — This appendix describes the parameters and functions used for modeling the infection dynamics of the chytrid fungus B. dendrobatidis in a population of the harlequin toad Atelopus cruciger. (ZIP) [file pone.0254439.s001.zip › S1_Appendix.pdf]

APPENDIX: Living with the pathogenic chytrid fungus: exploring mechanisms of coexistence in the harlequin toad *Atelopus cruciger.*

Onil Ballestas[1], Margarita Lampo[1*], Diego Rodríguez[2]

1 Centro de Ecología, Instituto Venezolano de Investigaciones Científicas, Caracas, Venezuela
2 Laboratorio de Ecología Evolutiva, Instituto de Ecología y Zoología Tropical, Facultad de Ciencias, Universidad Central de Venezuela, Caracas, Venezuela.

* mlampo@gmail.com

Abstract

This appendix describes the parameters and functions used in the article.

Methods

Description of model parameters and functions

The number of newborn tadpoles produced by an adult per week $f(t)$, was described by a cosine function with maximum value, f_{mx}, during the driest week of the year and a half period, S, defining the number of weeks during which the number of tadpoles produced is greater than zero (Fig. S1),

$$h(t) = f_{mx} - \left[\frac{f_{mx} - f_{mn}}{S - pl_{mn}}\right]\left[pl_{mx} - [\frac{pl_{mx} - pl_{mn}}{2}][1 - cos(\frac{2\pi}{52}(t - 10) + \pi)] - pl_{mn}\right]$$

$$\begin{aligned} f(t) &= h(t) \quad \text{for } h(t) \geq 0 \\ f(t) &= 0 \qquad \text{for } h(t) < 0 \end{aligned} \tag{A1}$$

where f_{mn} is the minimum fecundity. Maximum and minimum fecundities are in Table 1. Maximum and minimum rainfall are pl_{mx} and pl_{mn}, respectively.

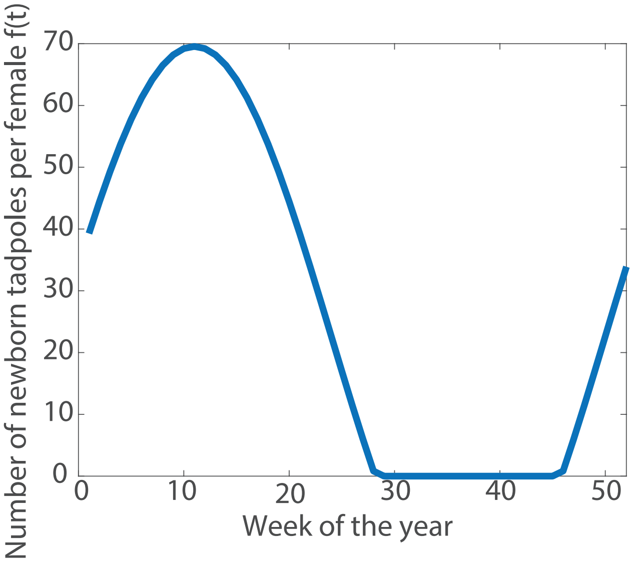

Figure 1. Tadpole recruitment function. Describes weekly variations in the per capita recruitment of new tadpoles into the population according to eqn. 1. Parameter values are described in Table 1. The seasonality parameter, S, defines the time span with $f(t) > 0$

Table 1. Parameter values used in the model and their bibliographic sources.

Parameter	Description	Value	Source
μ_A	Weekly per capita death rate of uninfected adults	0.07	[1]
μ_P	Weekly per capita death rate of uninfected tadpoles	0.07	unknown
μ_D	Density-dependent mortality in tadpoles	0.0004	unknown
g_A	Increase in adult mortality with the addition of a single zoospore estimated as the slope of a line with x-intercept at μ_A and y-intercept at lethal parasite load of 10^5 zoospores .	10^{-5}	[2, 3]
g_P	Increase in tadpole mortality with the addition of a single zoospore estimated as the slope of a line with x-intercept at μ_P and y-intercept at lethal parasite load of 10^5 zoospores.	0	unknown
$\upsilon\mu_E\beta_A$	Weekly per capita transmission rate from adults defined as the product of zoospore release, zoospore survival and zoospore acquisition	0.0125	[1]
$\upsilon\mu_E\beta_P$	Weekly per capita transmission rate from tadpoles defined as the product of zoospore release, zoospore survival and zoospore acquisition	0.0125	unknown
υ	Weekly fraction of zoospores released into the abiotic reservoir by a single infected adult	0.8	unknown
$b_Z - \mu_Z$	Weekly rate of increase of zoospores *in vitro*	2	[4]
$-\frac{1}{ln(1-\mu_E)}$	Life expectancy of a zoospore in the environment (weeks)	0.6	[4]
f_{mn}	Minimum number of newborns produced by an adult per week	45	[5]
f_{mx}	Maximum number of newborns produced by an adult per week	75	[5]
pl_{mn}	Maximum rainfall	200 mm	
pl_{mx}	Minimum rainfall	10 mm	

The per capita weekly rate of adult recruitment via maturation (Fig. S2) was defined by a three-step function where the per capita rate of maturation increased

exponentially between 9 and 12 weeks of age according to,

$$\begin{aligned} u(t) =& 0 \quad &\text{for} \quad & j \leq 8 \\ u(t) =& qe^{Bj} - r \quad &\text{for} \quad & 8 < j < 12 \\ u(t) =& 1 \quad &\text{for} \quad & j = 12 \end{aligned} \tag{A2}$$

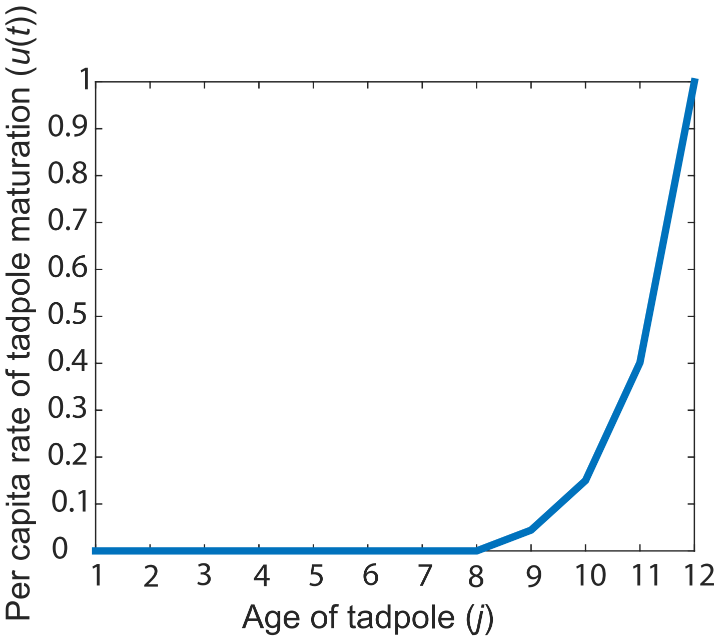

Figure 2. Tadpole maturation function. Describes weekly variations in the per capita maturation of new tadpoles into adults according to eqn. 2. Parameter values used were $q = 3.117x10^5$, B=0.8673, r=0.03214

The per capita rate of mortality of tadpoles $m_P(P(t), z_P(j,t))$ and adults $m_A(z_A(j,t))$ were defined by two-step linear functions,

$$q = \mu_P + \mu_D P + g_P z_P(j,t)$$

$$\begin{aligned} m_P(P(t), z_P(j,t)) = q \quad &\text{for} \quad & q < 1 \\ m_P(P(t), z_P(j,t)) = 1 \quad &\text{for} \quad & q \geq 1 \end{aligned} \tag{A3}$$

$$s = \mu_A + g_A z_A(j,t)$$

$$\begin{aligned} m_A(z_A(j,t)) = s \quad &\text{for} \quad & s < 1 \\ m_A(z_A(j,t)) = 1 \quad &\text{for} \quad & s \geq 1 \end{aligned} \tag{A4}$$

where μ_P and μ_A are the weekly per capita death rate of non-infected tadpoles and adults, μ_D is the increase in tadpole mortality by an addition of an accompanying tadpole and g_P and g_A denote the increase in the rate of per capita mortality of tadpoles and adults, respectively, by the infection with a single zoospore (Table 1).

References

1. Lampo M, Señaris C, García CZ. Population dynamics of the critically endangered toad *Atelopus cruciger* and the fungal disease chytridiomycosis. PLOS One. 2017;12(6):e0179007. doi:10.1371/journal.pone.0179007.

2. Langhammer PF, Lips KR, Burrowes PA, Tunstall T, Palmer CM, Collins JP. A fungal pathogen of amphibians, *Batrachochytrium dendrobatidis*, attenuates in pathogenicity with in vitro passages. PloS one. 2013;8(10):e77630.

3. Vredenburg VT, Knapp RA, Tunstall TS, Briggs CJ. Dynamics of an emerging disease drive large-scale amphibian population extinctions. Proceedings of the National Academy of Sciences of the United States of America. 2010;107(21):9689–9694.

4. Woodhams DC, Alford RA, Briggs CJ, Johnson M, Rollins-Smith LA. Life-history trade-offs influence disease in changing climates: Strategies of an amphibian pathogen. Ecology. 2008;89(6):1627–1639.

5. Castro N. Ecología del sapito arlequín de rancho grande *Atelopus cruciger* (Anura: Bufonidae) en el Río Cuyagua, Estado Aragua. Universidad Central de Venezuela Trabajo Especial de Grado Caracas, Venezuela. 2015;.
