# Supplementary material for: Living with the pathogenic chytrid fungus: Exploring mechanisms of coexistence in the harlequin toad Atelopus cruciger
Source: PLoS One. 2021 Jul 15;16(7):e0254439. doi: 10.1371/journal.pone.0254439 (PMC8281998; doi:10.1371/journal.pone.0254439)
Supplement: Attachment — Submitted filename: ResponseToReviewers.pdf [file pone.0254439.s003.pdf]

Response to Reviewers:

Line 3 *– This figure "500 amphibian species, including 90 presumed extinctions [1]." is not actually supported by sound evidence. See Lambert et al. 2020 response to the citation listed (DOI: 10.1126/science.aay1838). I recommend modifying this citation to omit these specific, inflated numbers or also cite the response as "[1], but see [Lambert et al. 2020]".*
We omitted figures and changed the first sentence to the more general statement: "Chytridiomycosis………has been linked to the local extirpation and extinction of amphibian populations worldwide, although the exact number of species affected remains controversial (Scheele et al 2019; Lambert et al.2020)". We directed the reader to the suggested references to enquire further about the estimated number of species affected.

Line 17 *– The citation for A. palmatus should be [13] not [12].*
Done

Line 25 *– Should be consistent in either using the abbreviated Bd or the longer B. dendrobatidis throughout the paper.*
We used *Bd* throughout the paper.

Figure 3 *– It would be helpful to include in the figure legend what the* colors correspond to.
I am not sure what the reviewer means. The figure legend reads: "Blue pixels represent combinations of parameters for which coexistence is the most likely outcome, green denotes pairs for which *Bd* is most likely to fail in establishing and red denotes pairs for which Bd is more likely to extirpate the toad population. Please let me know if you think we need to add more information.

Line 183 *– The opening statement of the discussion should have citations for the facts it is listing.*
We added seven references to the opening statement:
"The high impact of chytridiomycosis at high elevations (Catenazzi *et al.* 2014; Kriger & Hero 2008; Scheele *et al.* 2019; Woodhams & Alford 2005) and the recent discovery of recovering populations in lowlands (Grogan *et al.* 2016; Phillott *et al.* 2013; Rodríguez-Contreras *et al.* 2008) suggest that warm temperatures may promote the coexistence of some amphibian species with the pathogenic fungus *Bd* (Puschendorf *et al.* 2011; Zumbado-Ulate *et al.* 2014).

Line 195 *– Typo "estimated in" should be "estimated to be".*
Done

Line 218 *– The sentence "Harlequin toads tend to be short-lived." needs some qualifiers. For example, harlequin toads in captivity can live up to 10 years. The citations used imply that the authors mean to say that "Harlequin toads coexisting with Bd in the wild tend to be short-lived."*

We modified this sentence as follows: "Harlequin toads tend to be short-lived in the wild" Although in captivity harlequin toads can live in up to 10 years, mark-recapture studies suggest that in the wild uninfected adults of *A. cruciger* and *A. zeteki* have average life expectancies of less than one year. Thus, even in the absence of *Bd*, harlequin toads tend to be short-lived.

In general amphibians in captivity can last many years more than in the wild. I think that there are records of *R. marina* of about 40 years, but I doubt that they can live that long in the wild.

Line 222 *– The sentence "In the presence of a highly pathogenic Bd, only uninfected toads are likely to reproduce." Is there data to support this assertion? If so, you should cite here and perhaps explain in a bit more detail.*

There is no data demonstrating that infected toads cannot reproduce. However, given that they die within weeks after infection and amplexus in these toads can last months it seems unlikely that they can get infected and reproduce. I elaborated on this idea in the new version.

Line 224 *– There is a typo here. "when adults" should be "when individuals are adults."*
Done.

Line 238 *– The citation here is a review and should be cited as such (i.e., [reviewed in 36]).*
Done

Some seminal papers exploring Bd dynamics are not discussed or cited here and would add important context to this study.
For example Wilbur et al. 2017 (https://doi.org/10.1111/ele.12814) used a modelling approach to find that including an environmental zoospore pool increased R0 for R. muscosa-Bd systems. They also found that Bd-induced extinction risk was more sensitive to host resistance and tolerance than to the transmission dynamics of Bd. The similarities/differences between this study and that should be explored in the discussion.

Thanks for pointing out Wilbert et al.'s paper; it was enlightening. We mentioned their findings on the importance of resistance and tolerance on risk extinction in the Introduction section (lines X) Also, we included a whole paragraph explaining differences between these two models as I believe the comparison is highlight the need for context-specific modeling.

Additionally, the paper Vredenburg et al. 2010 (https://doi.org/10.1073/pnas.0914111107) is an important citation for estimating the lethal dose of Bd on an amphibian and should at least be mentioned or cited within given the estimation of lethal dose in this paper.
Vredenburg et al., 2010 was added to the list of sources of lethal doses used on Table 1 in the Appendix.
Further, the findings of Briggs et al. 2010 (https://doi.org/10.1073/pnas.0912886107) should be discussed in this paper. I think these papers should be included in the introduction and further explored in the discussion.
Briggs et al.'s paper was added to the list of papers supporting the importance of biotic or abiotic reservoirs in the extinction risk of host populations in the introduction and discussion sections.
