# Supplementary material for: Living with the pathogenic chytrid fungus: Exploring mechanisms of coexistence in the harlequin toad Atelopus cruciger
Source: PLoS One. 2021 Jul 15;16(7):e0254439. doi: 10.1371/journal.pone.0254439 (PMC8281998; doi:10.1371/journal.pone.0254439)
Supplement: Attachment — Submitted filename: ResponseToReviewers2.docx [file pone.0254439.s004.docx]

June 23, 2021

Stefan Lotters

Editor PlosOne

Dear Stefan Lötters,

Please find enclosed the second revision of the manuscript LIVING WITH THE PATHOGENIC CHYTRID FUNGUS: EXPLORING MECHANISMS OF COEXISTENCE IN THE HARLEQUIN TOAD ATELOPUS CRUCIGER for publication in *PlosOne* . All changes were made exactly as suggested by the referee. Below is the list of changes made (*in italics*) in the new version, in response to the reviewer’s suggestions.

Reviewer #1: I am satisfied with the changes made by the authors and feel that all comments have been adequately addressed. I especially like the new discussion paragraph discussing how the model describing this system compares to that built to describe the mountain yellow-legged frog system. I did notice a few typos/grammatical errors (mostly in the new text additions) that I will note below. Nice work!

**In the abstract:**

- replace "a disease produced by" with "a disease caused by"

*Replaced as suggested.*
- "most of the others are in high risk of extinction" should be "most of the others are at high risk of extinction"

*Replaced as suggested.*

**Line 267:** "have proved to be" should be "has proved to be"

*Replaced as suggested.*

**Line 271** **(and throughout this paragraph):** The common name for Rana muscosa is "Mountain Yellow-legged Frog" not "yellow-legged frog"

*Replaced as suggested.*

**Line 276:** The sentence that reads: "That is, not only recruiting health juveniles contribute to the susceptible population but also infected adults that have cleared infection." should be "That is, not only does the recruitment of healthy juveniles contribute to the susceptible population, but also the presence of infected adults that have cleared infection."

*Replaced as suggested.*

Once more, thanks for your support,

Sincerely,

Margarita Lampo
